# Transcriptomics, lifestyle, and autoimmune thyroiditis: an integrated association study

**DOI:** 10.3389/fimmu.2026.1853447

**Published:** 2026-06-24

**Authors:** Chun-Hu Li, Yu-Hang Liu, Zong-Yu Yue, Xiang-Kun Zeng, Ze-Xu Zhang, Xuan Li, Yi-Hang Liu, Ya-Hui Li, Tong Zhao, Peng Liu

**Affiliations:** Harbin Medical University, Center for Endemic Disease Control, Harbin, China

**Keywords:** autoimmune thyroiditis, immune-related genes, lifestyle behavior, RNA sequencing, transcriptomic risk score

## Abstract

**Background:**

Autoimmune thyroiditis (AIT) ranks among the most common autoimmune disorders globally. Adherence to a healthy lifestyle may substantially offset the risk conferred by a high transcriptomic risk profile.

**Methods:**

Immune-related differentially expressed genes (IRDEGs) were first identified based on data from the Gene Expression Omnibus (GEO) database (N_AIT_=13, N_non-AIT_=10) and a cross-sectional dataset from Anhui Province (N_AIT_=5, N_non-AIT_=5). Feature selection was then performed using LASSO regression and random forest algorithms to pinpoint key IRDEGs. These key genes were subsequently validated in a large, multi-province Chinese cross-sectional dataset (N = 902). Within this validation dataset, an RNA sequencing-based risk score (RSR score) was developed, and lifestyle factors were evaluated via structured questionnaires. The associations between the RSR score, lifestyle, and AIT were examined using multivariable logistic regression.

**Results:**

Through a multi-stage screening and validation process, three key genes were identified: IFI16, CASP4, and BKT. The study included 902 participants with a mean age of 39.17 ± 10.70 years, 56.32% of whom were female. Among them, 126 cases of AIT were identified (13.97%). The prevalence of AIT showed significant differences across strata of gender, lifestyle, and the RSR score. Logistic regression identified a high RSR score (OR = 2.58, 95% CI: 1.72–3.89), along with intermediate (OR = 2.38, 95% CI: 1.18–4.79) and unfavorable (OR = 2.84, 95% CI: 1.43–5.62) lifestyles, as significant risk factors for AIT. The robustness of these associations was confirmed in stratified analyses by age and gender.

**Conclusion:**

This study suggests independent and interactive roles of transcriptomic risk and lifestyle in AIT etiology. Adherence to a healthy lifestyle may substantially offset the risk conferred by high transcriptomic susceptibility.

## Introduction

Autoimmune thyroiditis (AIT) represents the most common organ-specific autoimmune disorder (1). Affecting approximately 3-5% of the global population, AIT has become one of the most prevalent autoimmune conditions (2). Notably, during the COVID-19 pandemic, clinical observations indicated that SARS-CoV-2 infection was not only associated with an increased incidence of AIT but could also trigger subacute thyroiditis or exacerbate pre-existing AIT (3–5).

The current clinical diagnosis of AIT primarily relies on serological testing, with the core criteria being the levels of thyroid peroxidase antibody (TPOAb) and thyroglobulin antibody (TgAb) (6). However, as significant antibody elevation often occurs after the disease has progressed subclinically for an extended period, current diagnostic strategies have limitations in the timely identification of AIT, potentially leading to delayed diagnosis for some individuals (7).

The etiology of AIT involves multifactorial interactions, including genetic susceptibility, environmental exposures, and their interplay at the transcriptomic level. Recent studies have highlighted the important role of Th17 cells and their secreted cytokines in the pathogenesis of AIT (8). Furthermore, transforming growth factor-beta contributes to the disease process through different signaling pathways, with its specific effects varying based on local concentration, individual genetic background, and disease stage (9). With the application of bioinformatics methods, Li et al. identified key genes and constructed predictive models, demonstrating promising potential for predicting AIT onset (7). RNA sequencing (RNA-Seq), a high-resolution transcriptome-wide analysis technique, provides a powerful tool for uncovering molecular signatures in diseased tissues (10). Researchers are increasingly leveraging RNA-Seq data to derive transcriptomic risk scores for enhanced predictive modeling (11). For instance, some studies have integrated single-cell and bulk RNA-Seq data with machine learning to build predictive models for rheumatoid arthritis (12). Similarly, He et al. developed an RNA-Seq-based risk score (RSR score) model to predict recurrence in papillary thyroid carcinoma (10).

This study integrates transcriptomic data from the Gene Expression Omnibus (GEO) with a multi-province cross-sectional survey in China to systematically evaluate the combined effects of transcriptomic and lifestyle factors on autoimmune thyroiditis (AIT) risk. It aims to identify key AIT-associated genes, construct a transcriptome-based risk score, and assess lifestyle factors via questionnaires, thereby elucidating their roles in AIT pathogenesis. This work seeks to identify high-risk populations and provide a scientific basis for early prevention and targeted interventions.

## Methods

### Study design and dataset overview

This study followed a three-stage design: discovery, feature selection, and population validation.

Discovery stage: Two public GEO transcriptomic datasets of thyroid tissue (GSE138198 and GSE54958) and one in-house peripheral blood RNA-seq dataset collected in Anhui Province in 2022 were integrated after batch effect correction. This combined dataset was used to identify immune-related differentially expressed genes (IRDEGs).

Feature selection stage: LASSO regression and random forest models were applied to the IRDEGs in the combined discovery set to select key genes.

Validation stage: Candidate gene expression was quantified by RT-qPCR in a multi-province cross-sectional cohort (n=902), and a RSR score was constructed based on the validated genes.

### Study population and sample collection

The cross-sectional survey covered six provinces (Qinghai, Shandong, Hebei, Anhui, Zhejiang, Yunnan). Four sampling sites (two urban, two rural) were selected per province, enrolling approximately 200 participants each. Inclusion criteria: age 18–60 years, local residence for at least three years. Exclusion criteria: (1) history of other acute or chronic autoimmune diseases; (2) use of antithyroid drugs or immunosuppressants within the past three months; (3) lactation or pregnancy.

Peripheral venous blood was collected from all participants. Serum was used to measure FT3, FT4, TSH, TgAb, and TPOAb. A portion of blood was stored at –80 °C for RNA extraction. AIT was defined by: (1) TgAb > 4 IU/mL and/or TPOAb > 9 IU/mL; (2) normal thyroid function; (3) no history of any thyroid disease. The protocol was approved by the Ethics Committee of Harbin Medical University, and all participants provided written informed consent.

### Reverse transcription polymerase chain reaction (RT-qPCR)

Total RNA was extracted from 902 validation samples using the TB Green^®^ Premix Ex Taq™ II kit (Takara, China). RNA quality was assessed by a NanoDrop 2000/2000C spectrophotometer (Thermo Fisher Scientific, USA), and qualified samples were reverse-transcribed into cDNA. Quantitative PCR was performed in 96-well plates using gene-specific primers and SYBR Green chemistry. The thermal cycling protocol consisted of an initial denaturation at 95 °C for 10 min, followed by 40 cycles of 95 °C for 15 s, primer-specific annealing for 30 s, and 72 °C for 30 s. Amplification specificity was confirmed by melt curve analysis. Gene expression was quantified using the 2^(-ΔCt) method with GAPDH as the endogenous control.

### Screening of immune-related differentially expressed genes (IRDEGs)

This study searched the GEO database for transcriptomic datasets from AIT patients. Datasets were included if they: (1) utilized thyroid tissue samples; (2) were intact and fully downloadable; (3) comprised an AIT group without other thyroid comorbidities; and (4) included a control group without any thyroid disease, including AIT. The discovery datasets comprised GSE138198 (13 AIT, 3 non-AIT; thyroid tissue), GSE54958 (7 non-AIT; thyroid tissue), and the Anhui case-control set (5 AIT, 5 non-AIT; peripheral blood RNA-seq). Each GEO dataset was first normalized with DESeq2 and variance-stabilizing transformation. Batch effects between the two GEO datasets were corrected using the ComBat function (sva R package), with principal component analysis confirming the correction. The Anhui RNA-seq data were similarly normalized with DESeq2. The corrected GEO data and the normalized Anhui data were then merged, and ComBat was applied again to remove potential residual batch and platform effects. The merged discovery set comprised 18 AIT cases and 15 non-AIT participants. In addition to the public GEO datasets, this study included an in-house dataset from a case-control study previously conducted in Anhui Province. Total RNA was extracted from the peripheral blood of these 10 participants using Trizol, and RNA-seq was performed on the DNBSEQ platform following standard protocols (BGI, Shenzhen, China).

Differential expression analysis between AIT patients and non-AIT participants was performed using the DESeq2 R package. Genes with an adjusted *P* < 0.05 and |log2(Fold Change)| ≥ 1 were defined as differentially expressed genes (DEGs) (13). By intersecting these DEGs with a list of experimentally validated immune-related genes obtained from the IMMPORT database, immune-related differentially expressed genes (IRDEGs) were identified.

### Feature selection using machine learning models

The identified IRDEGs from the integrated GEO and Anhui datasets underwent feature selection. First, a LASSO regression model was constructed to prevent overfitting and enhance interpretability (14). The model was built with AIT status as the outcome, and 10-fold cross-validation was used to select the optimal λ. Genes with non-zero coefficients were retained. The LASSO-selected genes were then used to build a random forest model, and five-fold cross-validation was again applied to estimate predictive performance and optimize hyperparameters (15). Model hyperparameters were optimized through five-fold cross-validation, and SHAP analysis was employed to quantify and visualize the contribution of each feature (16).

### Validation of IRDEGs and RSR score calculation

To validate the candidate genes in a large population, this study used the multi-province Chinese dataset. The LASSO-selected genes (IFI16, CASP4, and LY96) together with three additional genes from the literature (CCR1, IL1B, and BKT) (7, 17) were first examined. The Mann-Whitney U test was used to compare the expression of each gene between the AIT group and the non-AIT group. Genes that showed a statistically significant difference were then included in a multivariable logistic regression model. This model was adjusted for age, gender, ethnicity, annual per capita income, and serum iodine concentration. After adjustment, the regression coefficients (β) of the genes that remained significant were extracted. These β values were used to calculate the individual RSR score based on the following formula:


$$\text{RSR}=\sum_{i=1}^{n}(expr(Gene_{i})*coef(Gene_{i}))$$

Where *expr(Gene_i_)* represents the expression level of the *i*-th gene, and *coef(Gene_i_)* is its corresponding coefficient derived from the multivariable logistic regression model (10). In this study, the RSR score, as a continuous variable, was dichotomized into high and low groups based on its median value.

### Lifestyle behavior assessment

A composite lifestyle behavior score was constructed based on five modifiable factors: diet (including sugary beverages, dietary habits, cooking methods, taste preference, and fish consumption), moderate-to-vigorous physical activity, body weight (incorporating BMI and waist circumference), smoking status, and alcohol consumption. Each factor was assigned a binary score of 0 or 1, where 1 indicated a favorable lifestyle behavior. The total score was calculated as the sum of all five components, with higher scores reflecting a healthier overall lifestyle. Based on the total score, participants were classified into ‘Favorable,’ ‘Intermediate,’ and ‘Unfavorable’ groups(**Supplementary Table 1**) (18). In all subsequent analyses, the Favorable group served as the reference category.

### Statistical analysis

**Figure 1:**
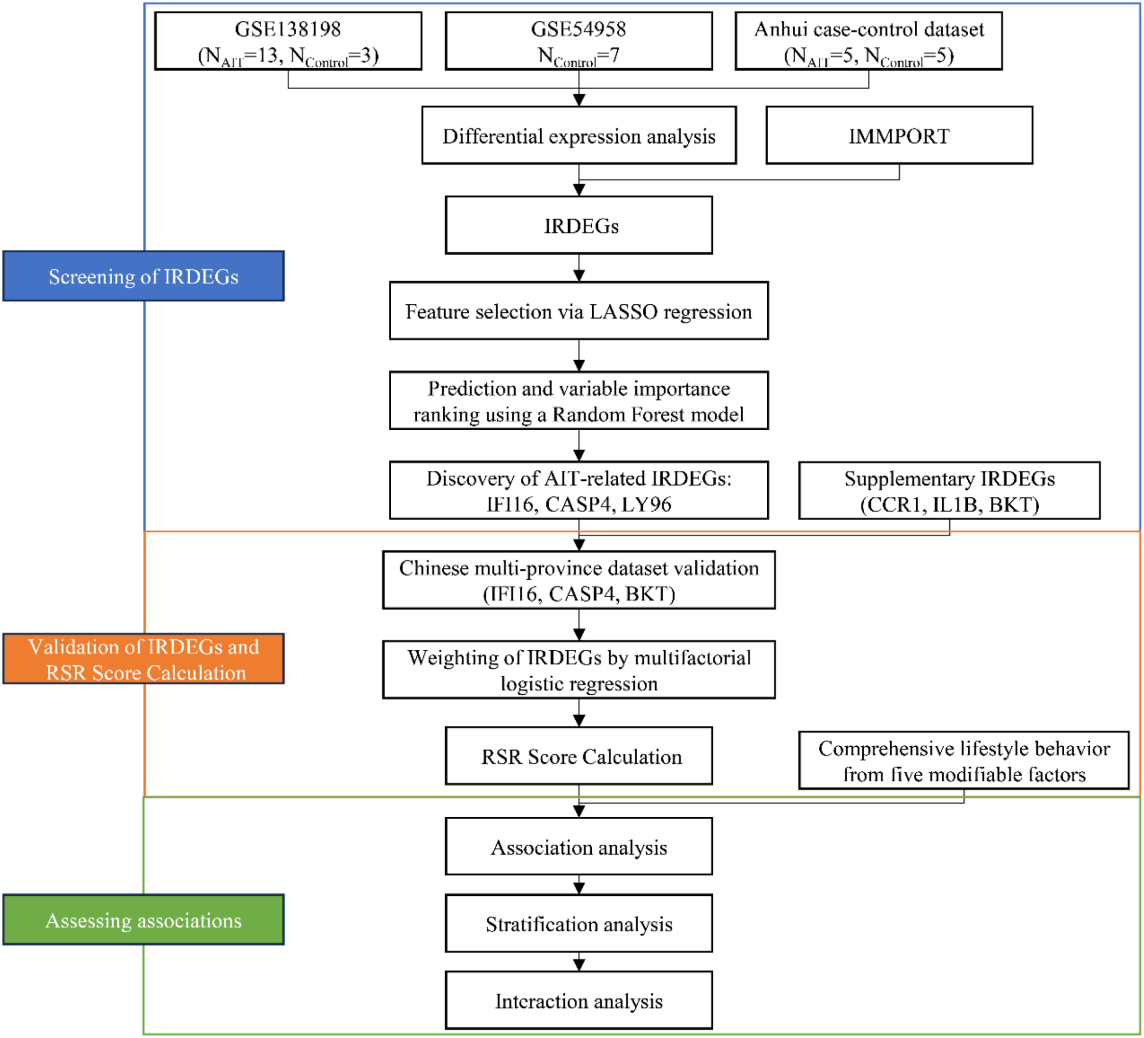
Overview of the research workflow.

Categorical variables are presented as frequencies and percentages, and continuous variables as mean ± standard deviation and median (Q_1_, Q_3_). Group comparisons were conducted using Pearson’s chi-square test or the Mann-Whitney U test. Logistic regression models were employed to assess the associations of lifestyle and RSR score with AIT, treating lifestyle as a continuous variable to test for trends. Two models were constructed: a crude model and an adjusted model controlling for age, gender, ethnicity, annual income per capita, and serum iodine concentration. Sensitivity analyses included stratification by age and gender. Multiplicative interaction between the RSR score and lifestyle was examined by including a product term in the regression model. All statistical analyses were performed using R software (version 4.4.1). The overall technical roadmap of this study is shown in **Figure 1**.

## Results

### Screening of IRDEGs based on multiple databases

**Figure 2:**
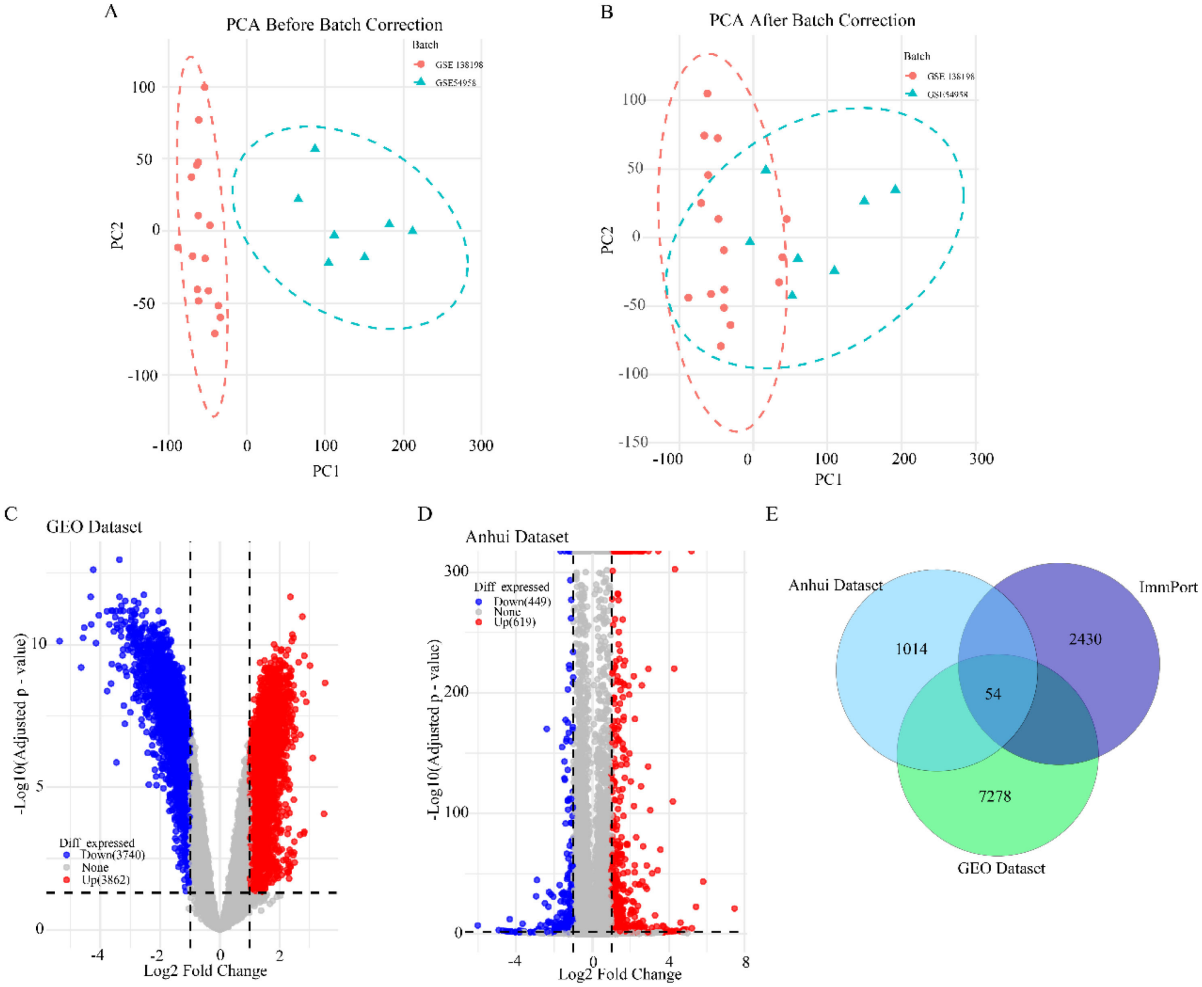
Integrated analysis and identification of DEGs from the GEO and Anhui transcriptomic datasets. **(A)**: Principal component analysis of the GSE138198 and GSE54958 datasets before batch effect correction. **(B)**: Principal component analysis of the GSE138198 and GSE54958 datasets after batch effect removal. **(C)**: Volcano plot of differentially expressed genes from the integrated GEO dataset (red: upregulated; blue: downregulated). **(D)**: Volcano plot of differentially expressed genes from the Anhui transcriptomic dataset (red: upregulated; blue: downregulated). **(E)**: Venn diagram showing the overlap of genes from the GEO dataset, Anhui transcriptomic dataset, and the immune gene set.

Prior to batch effect correction, principal component analysis of the two datasets from GEO revealed significant batch effects. After correction, the samples clustered more uniformly, allowing the datasets to be merged for subsequent analyses (**Figures 2A, B**). Differential expression analysis identified 7,602 DEGs (3,862 upregulated and 3,740 downregulated) in the integrated GEO dataset and 1,068 DEGs (619 upregulated and 449 downregulated) in the Anhui dataset (**Figures 2C, D**). Taking the intersection of these DEGs with a curated list of immune-related genes yielded 54 IRDEGs (**Figure 2E**).

**Figure 3:**
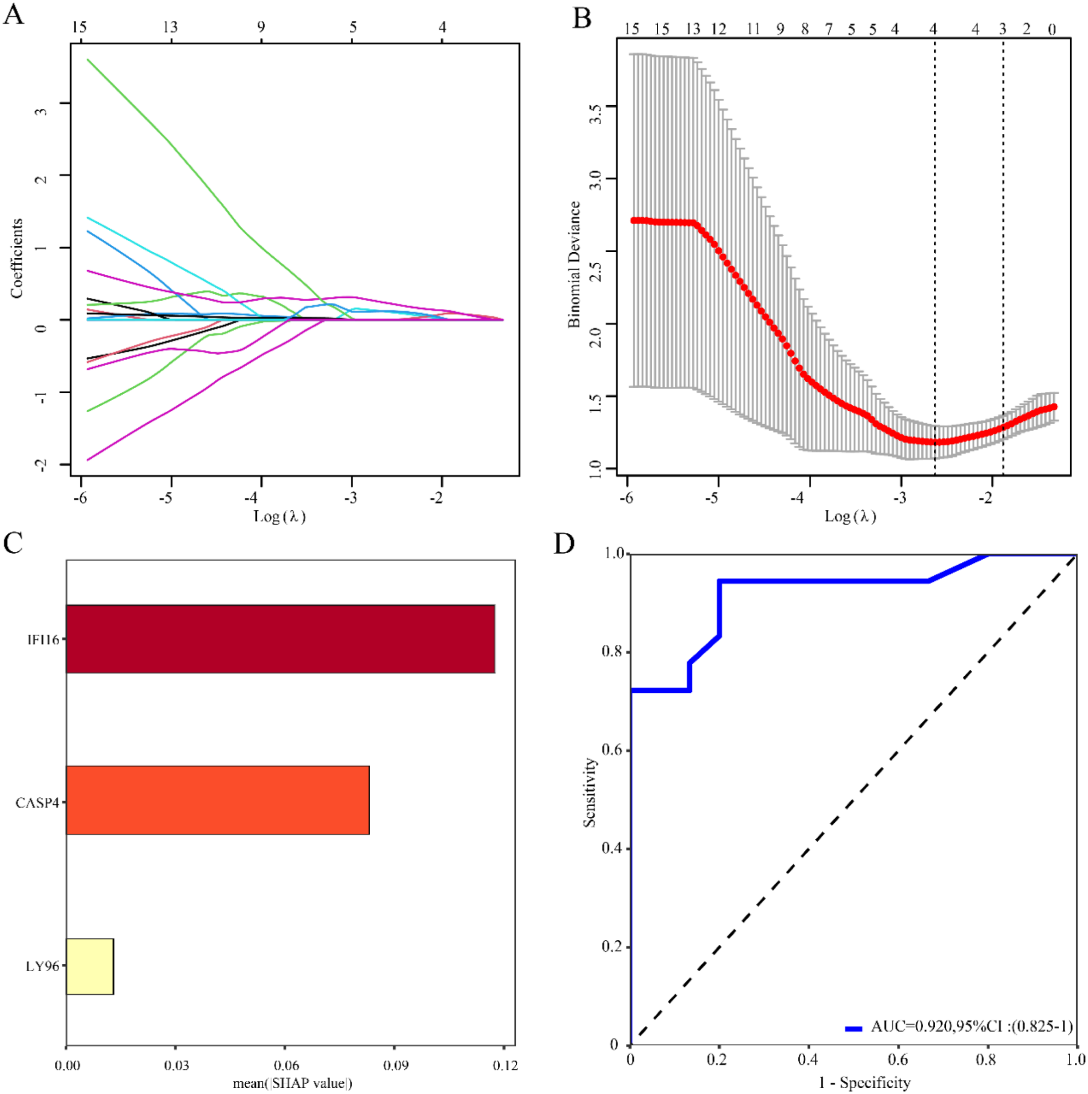
Gene selection based on LASSO regression and random forest. **(A)**: Profile of LASSO coefficient distribution. **(B)**: Cross-validation curve for tuning the LASSO penalty parameter. **(C)**: Feature importance ranking in the random forest model, as determined by SHAP values. **(D)**: Receiver operating characteristic curve of the random forest model constructed with the three key genes.

This study then integrated the GEO and Anhui datasets (N_AIT_=18, N_non-AIT_=15) and applied a lasso regression model to the 54 IRDEGs. This analysis identified three key IRDEGs: IFI16, CASP4, and LY96. A random forest model built using these genes demonstrated high predictive performance, with an area under the curve (AUC) of 0.92 (95% CI: 0.825-1.0). Variable importance analysis ranked IFI16 as the most influential predictor, followed by CASP4 and LY96 (**Figure 3**).

### Baseline characteristics of the multi-province dataset

Of 1,998 potential participants, 902 met the inclusion criteria (126 AIT patients and 776 non-AIT participants) (**Supplementary Figure 1**). Participants had a mean age of 39.17 years, were predominantly Han Chinese (82.26%), and 56.32% were female. Gender distribution differed significantly between AIT and non-AIT groups *(P* < 0.001). Based on lifestyle scores, participants were categorized into favorable (22.28%), intermediate (34.26%), and unfavorable (43.46%) groups, with significant differences in AIT prevalence across groups (P < 0.001). The overall mean RSR score was 0.04 ± 0.37, significantly higher in the AIT group (0.14 ± 0.35) than in the non-AIT group (0.03 ± 0.37) (**Table 1**).

**Table 1:** Baseline characteristics of the multi-province dataset.

Variables	Total (n = 902)	Non AIT (n = 776)	AIT (n = 126)	*P*
Age, Mean ± SD	39.17 ± 10.70	39.07 ± 10.84	39.79 ± 9.85	0.486
Race, n(%)				0.555
Han Chinese	742 (82.26)	636 (81.96)	106 (84.13)	
Others	160 (17.74)	140 (18.04)	20 (15.87)	
Annual Income Per Capita (CNY) (CNY), n(%)				0.587
<5000	196 (21.73)	169 (21.78)	27 (21.43)	
5000-10000	222 (24.61)	185 (23.84)	37 (29.37)	
10000-30000	178 (19.73)	158 (20.36)	20 (15.87)	
30000-50000	137 (15.19)	120 (15.46)	17 (13.49)	
>50000	169 (18.74)	144 (18.56)	25 (19.84)	
Gender, n(%)				<0.001
Female	508 (56.32)	408 (52.58)	100 (79.37)	
Male	394 (43.68)	368 (47.42)	26 (20.63)	
Lifestyle Behavior, n(%)				<0.001
Favorable	201 (22.28)	190 (24.48)	11 (8.73)	
Intermediate	309 (34.26)	265 (34.15)	44 (34.92)	
Unfavorable	392 (43.46)	321 (41.37)	71 (56.35)	
Physical activity, n(%)				0.009
Favorable	412 (45.68)	368 (47.42)	44 (34.92)	
Unfavorable Favorable	490 (54.32)	408 (52.58)	82 (65.08)	
Healthy weight, n(%)				0.950
Favorable	463 (51.33)	398 (51.29)	65 (51.59)	
Unfavorable Favorable	439 (48.67)	378 (48.71)	61 (48.41)	
Drinking, n(%)				0.100
Favorable	595 (65.96)	520 (67.01)	75 (59.52)	
Unfavorable Favorable	307 (34.04)	256 (32.99)	51 (40.48)	
Smoking, n(%)				0.639
Favorable	672 (74.50)	576 (74.23)	96 (76.19)	
Unfavorable Favorable	230 (25.50)	200 (25.77)	30 (23.81)	
Diet, n(%)				0.081
Favorable	787 (87.25)	671 (86.47)	116 (92.06)	
Unfavorable Favorable	115 (12.75)	105 (13.53)	10 (7.94)	
RSR score, M(Q_25_~Q_75_)	0.10 (-0.09, 0.25)	0.08 (-0.10, 0.24)	0.20 (0.00, 0.33)	0.002
RSR score, n(%)				<0.001
Low RSR score	451 (50.00)	409 (52.71)	42 (33.33)	
High RSR score	451 (50.00)	367 (47.29)	84 (66.67)	

**Supplementary Table 2** summarizes the thyroid function markers. Briefly, FT3, TSH, TG, TPOAb, and TGAb differed significantly between groups (all *P* < 0.05), whereas FT4 and serum iodine did not. FT3 was lower in the AIT group (median 4.81 vs. 4.96 pmol/L, *P* = 0.007). TSH was higher in the AIT group (2.43 vs. 2.11 mIU/L, *P* = 0.003). TPOAb and TGAb were markedly elevated in the AIT group (both *P* < 0.001).

### Gene validation

**Figure 4:**
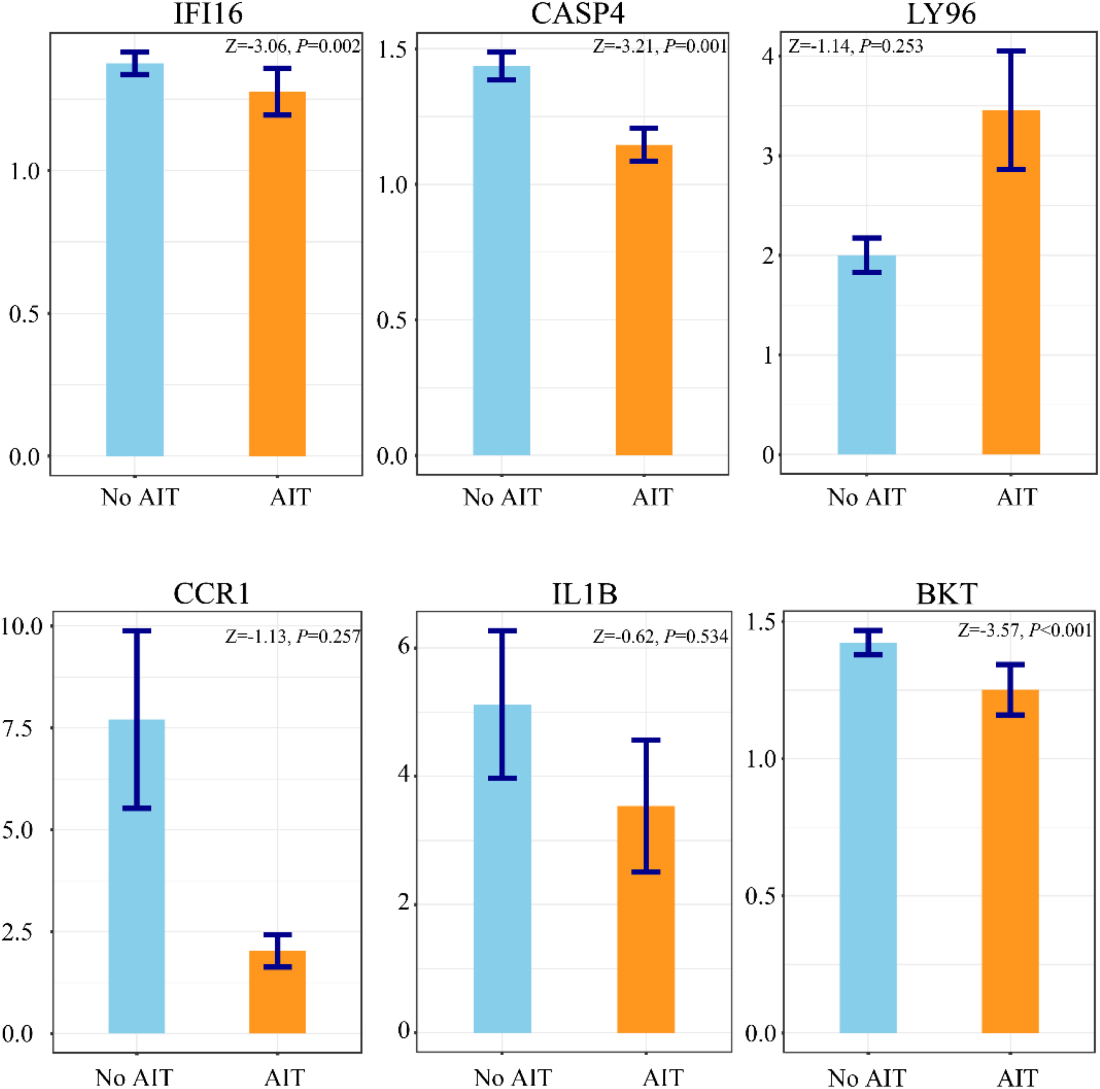
Validation of the expression levels of the screened genes in the multi-province cross-sectional cohort from China.

RT-qPCR analysis was performed on three previously identified IRDEGs (IFI16, CASP4, LY96) in 902 participants to validate their differential expression between AIT patients and non-AIT participants. Furthermore, to explore additional potential biomarkers, three other genes (CCR1, IL1B, and BKT) were selected based on a literature review for their suspected relevance to AIT. The results confirmed that IFI16, CASP4, and BKT exhibited statistically significant differential expression between the AIT and control groups (**Figure 4**).

### RSR score and lifestyle behavior profiles

A multivariate logistic regression model constructed using IRDEGs revealed that only IFI16 showed significant association with AIT in unadjusted analysis. After covariate adjustment, both IFI16 (β = -2.39, *P* = 0.017) and BKT (β = 0.34, *P* = 0.036) remained significant(**Supplementary Table 3**). The RSR score derived from these β-coefficients and expression data demonstrated significant distributional differences between case and non-AIT groups (*P* < 0.001) (**Table 1**).

The composite lifestyle behavior variable showed unequal distribution across categories (Favorable: 22.28%; Unfavorable: 43.46%), with significant differential distribution of AIT cases across lifestyle strata (*P* < 0.001) (**Table 1**).

### Association analysis

Spearman correlation analysis revealed significant negative correlations between AIT and male gender (r = -0.19, *P* < 0.001), physical activity (r = -0.09, *P* < 0.01), and expression of IFI16 (r = -0.16, *P* < 0.001), CASP4 (r = -0.15, *P* < 0.001), and BKT (r=-0.09, *P* < 0.01). Positive correlations emerged with lifestyle behavior (r = 0.13, *P* < 0.001) and RSR score (r = 0.15, *P* < 0.001). Strong intercorrelations were observed among the three key genes, while lifestyle behavior primarily correlated with physical activity, smoking, and diet patterns(**Supplementary Figure 2**).

Logistic regression models demonstrated significant associations in unadjusted analyses between high RSR score (vs low) and AIT (OR = 2.28, 95%CI:1.53-3.40), and between intermediate/unfavorable lifestyle behavior (vs favorable) and AIT (OR = 2.94, 95%CI: 1.47-5.86; OR = 3.92, 95%CI: 2.02-7.61). These associations persisted after covariate adjustment: high RSR score (OR = 2.63, 95%CI: 1.74-3.97); intermediate lifestyle behavior (OR = 2.43, 95%CI: 1.20-4.93); unfavorable lifestyle behavior (OR = 2.95, 95%CI: 1.48-5.88). Trend tests confirmed increasing AIT risk with deteriorating lifestyle patterns(**Table 2**).

**Table 2:** Associations of lifestyle behavior and RSR score with AIT.

Variables	Model 1^*^		Model 2^#^	
	OR(95%CI)	*P* value	OR(95%CI)	*P* value
RSR score	2.56 (1.42 ~ 4.61)	0.002	3.31 (1.74 ~ 6.30)	<0.001
Low	Ref			
High	2.28 (1.53 ~ 3.40)	<0.001	2.63 (1.74 ~ 3.97)	<0.001
Lifestyle Behavior				
Favorable	Ref			
Intermediate	2.94 (1.47 ~ 5.86)	0.002	2.43 (1.20 ~ 4.93)	0.014
Unfavorable	3.92 (2.02 ~ 7.61)	<0.001	2.95 (1.48 ~ 5.88)	0.002
Trend test	*P* < 0.001			

*Model 1 was not adjusted;.

#Model 2 was adjusted for age, gender, race, annual income per capita, serum iodine concentration.

Separate analysis of RSR score identified significant association between high RSR score and AIT after adjustment (OR = 2.58, 95%CI: 1.72-3.89) (**Supplementary Table 4**). Similarly, both intermediate and unfavorable lifestyle behavior showed significant positive associations with AIT (OR = 2.38, 95%CI: 1.18-4.79; OR = 2.84, 95%CI: 1.43-5.62). Analysis of individual lifestyle behavior components identified physical activity (OR = 1.58, 95%CI: 1.05-2.37) and alcohol consumption (OR = 1.58, 95%CI: 1.05-2.39) as significant independent factors(**Supplementary Table 5**).

### Stratified analysis

RSR score-stratified analysis revealed no significant lifestyle-AIT association in the low-RSR score stratum. However, in high-RSR score individuals, both intermediate (OR = 2.57, 95%CI: 1.05-6.29) and unfavorable lifestyle behavior (OR = 3.40, 95%CI: 1.42-8.10) significantly increased AIT risk compared to favorable lifestyle behavior (**Table 3**). Sensitivity analyses stratified by gender and age confirmed robust associations across all subgroups. Consistent associations were observed in both age strata (≤45 and >45 years) (**Table 4**).

**Table 3:** Associations between lifestyle behaviors with AIT, stratified by RSR score levels.

Variables	Model 1^*^		Model 2^*^	
	OR(95%CI)	*P* value	OR(95%CI)	*P* value
Low RSR				
Lifestyle Behavior				
Favorable	Ref			
Intermediate	2.53 (0.81 ~ 7.84)	0.109	2.17 (0.68 ~ 6.92)	0.191
Unfavorable	3.16 (1.06 ~ 9.40)	0.039	2.31 (0.74 ~ 7.21)	0.148
High RSR				
Lifestyle Behavior				
Favorable	Ref			
Intermediate	3.17 (1.33 ~ 7.56)	0.009	2.57 (1.05 ~ 6.29)	0.038
Unfavorable	4.40 (1.91 ~ 10.13)	<0.001	3.40 (1.42 ~ 8.10)	0.006

*Model 1 was not adjusted;.

#Model 2 was adjusted for age, gender, race, annual income per capita, serum iodine concentration.

**Table 4:** Associations of genetic risk and lifestyle with AIT, stratified by gender and age.

Variables	Model 1^*^		Model 2^*^	
	OR(95%CI)	*P* value	OR(95%CI)	*P* value
Male				
RSR score				
Low	Ref			
High	2.34 (0.95 ~ 5.73)	0.064	3.35 (1.52 ~ 5.25)	0.027
Lifestyle Behavior				
Favorable	Ref			
Intermediate	2.83 (0.87 ~ 9.16)	0.082	2.67 (0.80 ~ 8.88)	0.110
Unfavorable	3.13 (0.96 ~ 10.16)	0.057	3.06 (1.03 ~ 10.40)	0.034
Female				
RSR score				
Low	Ref			
High	2.61 (1.65 ~ 4.13)	<0.001	2.70 (1.69 ~ 4.30)	<0.001
Lifestyle Behavior				
Favorable	Ref			
Intermediate	2.09 (0.87 ~ 5.03)	0.101	2.09 (0.85 ~ 5.10)	0.106
Unfavorable	2.51 (1.08 ~ 5.83)	0.033	2.63 (1.11 ~ 6.22)	0.028
≤45				
RSR score	2.39 (1.46 ~ 3.89)	<0.001	2.79((1.69 ~4.62)	<0.001
Low	Ref			
High				
Lifestyle Behavior				
Favorable	Ref			
Intermediate	2.47 (1.08 ~ 5.63)	0.031	2.01(0.86~4.66)	0.106
Unfavorable	3.19 (1.46 ~ 6.97)	0.004	2.41 (1.07~5.43)	0.034
>45				
RSR score				
Low	Ref			
High	2.11 (1.04 ~ 4.27)	0.038	2.29(1.09~4.83)	0.029
Lifestyle Behavior				
Favorable	Ref			
Intermediate	4.21 (1.17 ~ 15.11)	0.028	3.00(0.79~11.31)	0.105
Unfavorable	6.57 (1.86 ~ 23.15)	0.003	4.56(1.23~16.87)	0.023

*Model 1 was not adjusted;.

#Model 2 was adjusted for race, annual income per capita, serum iodine concentration.

### Interaction effects

Significant multiplicative interaction was detected between RSR score and lifestyle. In **Supplementary Table 6**, compared to the high-RSR score/unfavorable-lifestyle behavior reference group, high-RSR individuals with favorable lifestyle showed substantially reduced AIT risk (OR = 0.28, 95%CI: 0.11-0.64). All combinations containing low RSR score demonstrated protective effects relative to the reference.

### Predictive performance

Four predictive models revealed incremental improvement in discriminative ability: the baseline model (adjusted for age, race, annual income per capita, serum iodine concentration) achieved AUC = 0.66; addition of RSR score or lifestyle behavior separately increased AUC to 0.70 and 0.69 respectively; the full model incorporating both RSR score and lifestyle behavior attained maximum predictive accuracy (AUC = 0.72, 95%CI: 0.67-0.76) (**Supplementary Figure 3**). Nomogram analysis identified gender, RSR score, and lifestyle behavior as primary predictive contributors, with RSR score demonstrating the highest relative importance(**Supplementary Figure 4**).

## Discussion

This study constructed an RSR score from RNA-Seq data to quantify transcriptomic risk and assessed lifestyle through questionnaires. Analysis showed that both unfavorable lifestyle and high RSR score independently increase AIT risk, with the highest risk observed when both are present. This aligns with studies on conditions like thyroid cancer, where high transcriptomic or polygenic risk combined with adverse lifestyle factors significantly elevates disease risk (18).

AIT represents a highly prevalent chronic autoimmune disorder and is the leading cause of hypothyroidism (19). Its diagnostic criteria remain somewhat ambiguous, and reliance solely on TgAb and TPOAb levels, while overlooking genetic predispositions, can lead to delayed or missed diagnoses (20). This study therefore sought to identify key genes associated with AIT. Initial analysis identified potential genes, including IFI16, CASP4, and LY96. To enhance the robustness of the findings, these were integrated with previously reported AIT-related immune genes (such as CCR1 and IL1B identified by Li et al., and BKT reported by Liu et al.) into a random forest model. This model, based on IFI16, CASP4, and LY96, demonstrated high predictive performance. Subsequent validation in a large cross-sectional dataset confirmed IFI16, CASP4, and BKT as key genes.

RNA-Seq technology provides a valuable resource for deciphering the molecular characteristics of disease and has shown significant progress in risk stratification models for conditions such as lung adenocarcinoma (21, 22) and asthma (11). Drawing on this approach, an RSR score was constructed based on the identified key genes. After multivariable adjustment, the RSR score showed a significant association with AIT risk, which remained stable after stratification by age and gender, confirming its robustness. Furthermore, incorporating the RSR score into predictive models enhanced their performance, suggesting that the RSR score may serve as a useful predictive indicator for AIT. However, given the moderate discriminative ability of the full model and the lack of external validation, further evaluation in independent cohorts is warranted before the clinical utility of this score can be established.

Regarding lifestyle assessment, this study integrated multiple dimensions-physical activity, smoking, alcohol consumption, diet, and healthy weight-providing a more comprehensive understanding of its relationship with AIT compared to single metrics. Previous research has confirmed that favorable lifestyle behavior is closely related to the onset, progression, and prognosis of autoimmune diseases (23). For instance, Western dietary patterns are associated with various autoimmune conditions, while modifying factors like smoking and stress can optimize personalized treatment for AIT (24, 25). This study specifically identified significant associations between AIT risk and both physical activity and alcohol consumption. Existing literature indicates that physical activity is significantly correlated with thyroid function markers (TSH, T3, T4), and its effects on Hashimoto’s thyroiditis may differ between occupational and leisure-time activities, potentially mediated through immune pathways such as cytokine regulation (1, 26). Concerning alcohol consumption, some studies have reported an inverse correlation between drinking frequency and Hashimoto’s thyroiditis risk (27), although chronic excessive intake may also disrupt thyroid function, manifesting as elevated TSH and FT4 levels (28). Although the present study did not find significant associations for diet, body weight, or smoking with AIT, existing literature suggests that smoking may indirectly affect the thyroid by reducing thyroid antibody levels (29), while obesity (high BMI) has been shown to increase the risk of positive thyroid antibodies, particularly in older populations (30).

Most diseases arise from the combined effects of transcriptomic regulation, lifestyle, and environmental factors (31). Stratified analysis by RSR score revealed that the effect of lifestyle on AIT risk was modified by transcriptomic background, with lifestyle being significantly associated with AIT only in the high RSR score group. This suggests that the association between modifiable lifestyle factors and AIT becomes clinically relevant primarily in individuals with a high RSR score. This pattern is consistent with the important role of gene expression profiles in AIT etiology. The observed synergistic association between a high RSR score and adverse lifestyle parallels findings in other complex diseases, underscoring a common pathogenic interaction (18, 32, 33). Assessment of a multiplicative interaction term revealed that compared to the highest-risk reference group (high transcriptomic risk score + unfavorable lifestyle), the combination of a low transcriptomic risk score and favorable lifestyle offered the strongest protective effect. Notably, even among individuals with a low RSR score, those with unfavorable lifestyle behavior still showed a significantly higher odds of AIT than their counterparts with favorable lifestyles. These results suggest that AIT prevention and control strategies should not only focus on high-risk individuals but also emphasize active modification of unfavorable lifestyle behaviors. However, the statistical interaction observed between the RSR score and lifestyle behavior warrants further investigation to determine whether it reflects true biological synergism.

Several limitations of this study should be acknowledged. First, the cross-sectional design prevents the determination of causal relationships. Reverse causation is also possible: having AIT may change a person’s lifestyle behaviors (such as physical activity or diet) or affect their gene expression profiles. Therefore, we cannot rule out that the observed associations are partly driven by the disease itself rather than the other way around. Second, although environmental factors are known to influence AIT development, relevant environmental exposure data were not available in the present study. Third, although the study enrolled 902 participants, larger prospective cohorts are needed to further validate these findings. Fourth, the discovery datasets differed in sample source (thyroid tissue and peripheral blood), analytical platform, and sample size. This heterogeneity may affect the identification of differentially expressed genes. However, batch effect correction was applied and the key genes were validated in a large independent population, which partially mitigates this concern.

## Conclusion

This study suggests that a healthy lifestyle is associated with a lower odds of AIT, even among individuals with a high RSR score. Therefore, lifestyle intervention may represent a cost-effective preventive strategy, particularly for individuals with high transcriptomic risk profile.

## Data Availability

The raw data supporting the conclusions of this article will be made available by the authors, without undue reservation.
