# Supplementary material for: Transcriptomics, lifestyle, and autoimmune thyroiditis: an integrated association study
Source: Front Immunol. 2026 Jun 24;17:1853447. doi: 10.3389/fimmu.2026.1853447 (PMC13341861; doi:10.3389/fimmu.2026.1853447)
Supplement: Supplementary file 1 [file Table1.docx]

**Transcriptomics, Lifestyle, and Autoimmune Thyroiditis: An Integrated Association Study**

Chun-Hu Li^1^, Yu-Hang Liu^1^, Zong-Yu Yue^1^, Xiang-Kun Zeng^1^, Ze-Xu Zhang^1^, Xuan Li^1^, Yi-Hang Liu^1^, Ya-Hui Li^1^, Tong Zhao^1^, Peng Liu^1^*

1. Harbin Medical University, Center for Endemic Disease Control, Harbin, China,150081.

* Corresponding author

Email: liup7878@163.com (PL)

**Contents**

**TableS1.** Healthy lifestyle components.

**TableS2.** Associations of IRDEGs with AIT via Multivariable Logistic Regression.

**TableS3.** Thyroid Function Profiles in the Multi-Province Cross-Sectional dataset.

**TableS4.** Association Between RSR Score and AIT.

**TableS5.** Associations of Multiple Lifestyle Behaviors with AIT.

**TableS6.** Interaction Effects Between Genetic Risk and Lifestyle on AIT.

**FigureS1.** Participant Flowchart for the Multi-Province Cross-Sectional Study.

**FigureS2.** Correlation Analysis of Baseline Characteristics, IRDEGs, RSR Score, and Lifestyle Behavior with AIT.

**FigureS3.** Comparison of Predictive Performance Across Variables Using Logistic Regression.

**FigureS4.** Nomogram Derived from the Logistic Regression Model.

**TableS1.** Healthy lifestyle components.

| Healthy lifestyle factors | Source and definition | Score |
| --- | --- | --- |
| 1. Total moderate-vigorous physical activity | >=150 minutes moderate activity per week or >= 75 minutes vigorous activity per week | Favorable |
|  | >=150 minutes moderate activity per week or >= 75 minutes vigorous activity per week | Unfavorable |
| 2. Be a Weight | BMI (kg/m^2^) |  |
|  | 18.5-24.9 | Favorable |
|  | 25-29.9 | Intermediate |
|  | ≥30 or <18.5 | Unfavorable |
|  | Waist circumference (cm) |  |
|  | Men: <94 | Favorable |
|  | Women: <80 |  |
|  | Men: 94-<102 | Intermediate |
|  | Women: 80-<88 |  |
|  | Men: >=102 | Unfavorable |
|  | Women: >=88 |  |
|  | Weight |  |
|  | The sum score of BMI and waist circumference >=4 | Favorable |
|  | The sum score of BMI and waist circumference < 4 | Unfavorable |
| 3. Diet | sugar-sweetened beverage consumption =0 servings/week |  |
|  | Fish: >=3 servings/week |  |
|  | Primary cooking method: Boiling, Steaming, Making cold dishes, Stir-frying |  |
|  | Dietary preference: Prefers salty food, Other |  |
|  | Dietary taste preference: Prefers mild or moderate flavors |  |
|  | Dietary pattern: Omnivorous (balanced) |  |
|  | Adequate intake of >=3 dietary components | Favorable |
|  | Adequate intake of <3 dietary components | Unfavorable |
| 4. Smoke | Never and previous | Favorable |
|  | Current | Unfavorable |
| 5. Drink | Never and previous | Favorable |
|  | Current | Unfavorable |
| Lifestyle | 3-5 favorable lifestyle factors | Favorable |
|  | 2 favorable lifestyle factors | Intermediate |
|  | 0-1 favorable lifestyle factors | Unfavorable |

**TableS2.** Thyroid Function Profiles in the Multi-Province Cross-Sectional dataset.

| Variables | Total  (n = 902) | Non AIT  (n = 776) | AIT  (n = 126) | *P* |
| --- | --- | --- | --- | --- |
|  |  |  |  |  |
| FT3 (pmol/L), M (Q₁, Q₃) | 4.93 (4.54, 5.39) | 4.96 (4.56, 5.42) | 4.81 (4.40, 5.16) | 0.007 |
| FT4 (pmol/L), M (Q₁, Q₃) | 11.20 (9.97, 12.40) | 11.23 (9.98, 12.42) | 11.02 (9.80, 12.36) | 0.130 |
| TSH (mIU/L), M (Q₁, Q₃) | 2.16 (1.51, 3.06) | 2.11 (1.49, 2.94) | 2.43 (1.64, 3.71) | 0.003 |
| TG (mmol/L), M (Q₁, Q₃) | 7.54 (4.44, 11.68) | 7.95 (5.03, 11.77) | 1.78 (0.35, 9.71) | <0.001 |
| TPOAb (IU/ml), M (Q₁, Q₃) | 0.77 (0.39, 1.92) | 0.65 (0.36, 1.28) | 53.17 (10.26, 179.76) | <0.001 |
| TGAb (IU/ml), M (Q₁, Q₃) | 0.14 (0.10, 0.24) | 0.13 (0.09, 0.18) | 11.59 (1.57, 56.57) | <0.001 |
| Serum Iodine (umol/L), M (Q₁, Q₃) | 65.43 (53.22, 79.96) | 64.85 (53.15, 79.55) | 67.64 (54.75, 81.13) | 0.538 |

**TableS3.** Associations of IRDEGs with AIT via Multivariable Logistic Regression.

| Gene | Model 1^*^ | | Model 2^#^ | |
| --- | --- | --- | --- | --- |
|  | *β* | *P* | *β* | *P* |
| IFI16 | -2.20 | 0.024 | -2.39 | 0.017 |
| CASP4 | -0.48 | 0.086 | -0.47 | 0.098 |
| BKT | 0.21 | 0.182 | 0.34 | 0.036 |

*: Model 1 was not adjusted;

#: Model 2 was adjusted for age, gender, race, annual income per capita, serum iodine concentration.

**TableS4.** Association Between RSR Score and AIT.

| Variables | Model 1^*^ | | Model 2^#^ | |
| --- | --- | --- | --- | --- |
|  | OR(95%CI) | *P* value | OR(95%CI) | *P* value |
| RSR score | 2.56 (1.42 ~ 4.61) | 0.002 | 3.27 (1.73 ~ 6.17) | <0.001 |
| Low | Ref | | | |
| High | 2.23 (1.50 ~ 3.31) | <0.001 | 2.58 (1.72 ~ 3.89) | <0.001 |

*: Model 1 was not adjusted;

#: Model 2 was adjusted for age, gender, race, annual income per capita, serum iodine concentration.

**TableS5.**Associations of Multiple Lifestyle Behaviors with AIT.

| Variables | Model 1^*^ | | Model 2^*^ | |
| --- | --- | --- | --- | --- |
|  | OR(95%CI) | *P* value | OR(95%CI) | *P* value |
| Lifestyle Behavior |  |  |  |  |
| Favorable | Ref | | | |
| Intermediate | 2.87 (1.44 ~ 5.70) | 0.003 | 2.38 (1.18 ~ 4.79) | 0.015 |
| Unfavorable | 3.82 (1.97 ~ 7.39) | <0.001 | 2.84 (1.43 ~ 5.62) | 0.003 |
| Physical activity |  |  |  |  |
| Favorable | Ref | | | |
| Unfavorable | 1.66 (1.12 ~ 2.47) | 0.012 | 1.58 (1.05 ~ 2.37) | 0.029 |
| Diet |  |  |  |  |
| Favorable | Ref | | | |
| Unfavorable | 1.72 (0.87 ~ 3.42) | 0.120 | 1.57 (0.78 ~ 3.16) | 0.207 |
| Healthy weight |  |  |  |  |
| Favorable | Ref | | | |
| Unfavorable | 0.96 (0.65 ~ 1.40) | 0.818 | 0.94 (0.63 ~ 1.39) | 0.748 |
| Smoke |  |  |  |  |
| Favorable | Ref | | | |
| Unfavorable | 1.13 (0.71 ~ 1.78) | 0.613 | 1.26 (0.79 ~ 2.01) | 0.333 |
| Drinking |  |  |  |  |
| Favorable | Ref | | | |
| Unfavorable | 0.70 (0.47 ~ 1.04) | 0.080 | 1.58 (1.05 ~ 2.39) | 0.028 |

*: Model 1 was not adjusted;

#: Model 2 was adjusted for age, gender, race, annual income per capita, serum iodine concentration.

**TableS6.** Interaction Effects Between Genetic Risk and Lifestyle on AITD.

| Variables | | Model 1^*^ | | Model 2^*^ | |
| --- | --- | --- | --- | --- | --- |
| RSR | Lifestyle Behavior | OR(95%CI) | *P* value | OR(95%CI) | *P* value |
| High | Unfavorable | Ref | | | |
| High | Intermediate | 0.72 (0.43 ~ 1.21) | 0.217 | 0.75 (0.44 ~ 1.28) | 0.298 |
| High | Favorable | 0.23 (0.09 ~ 0.49) | <0.001 | 0.28 (0.11 ~ 0.64) | 0.004 |
| Low | Unfavorable | 0.41 (0.23 ~ 0.70) | 0.001 | 0.33 (0.19 ~ 0.57) | <0.001 |
| Low | Intermediate | 0.33 (0.17 ~ 0.60) | <0.001 | 0.32 (0.16 ~ 0.59) | <0.001 |
| Low | Favorable | 0.13 (0.04 ~ 0.45) | <0.001 | 0.15 (0.04 ~ 0.40) | <0.001 |

*: Model 1 was not adjusted;

#: Model 2 was adjusted for age, gender, race, annual income per capita, serum iodine concentration.

**
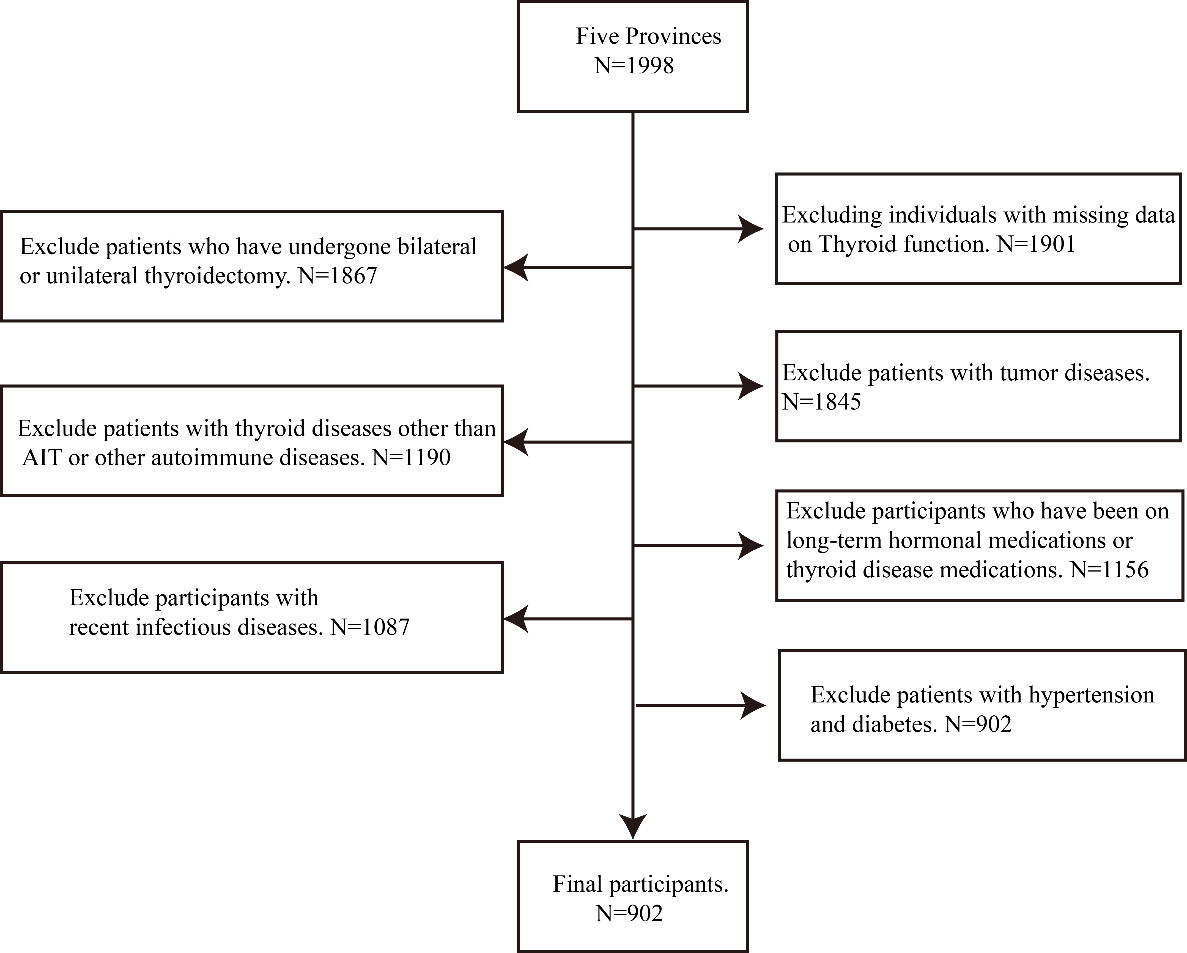
**

**FigureS1.**Participant Flowchart for the Multi-Province Cross-Sectional Study.

**
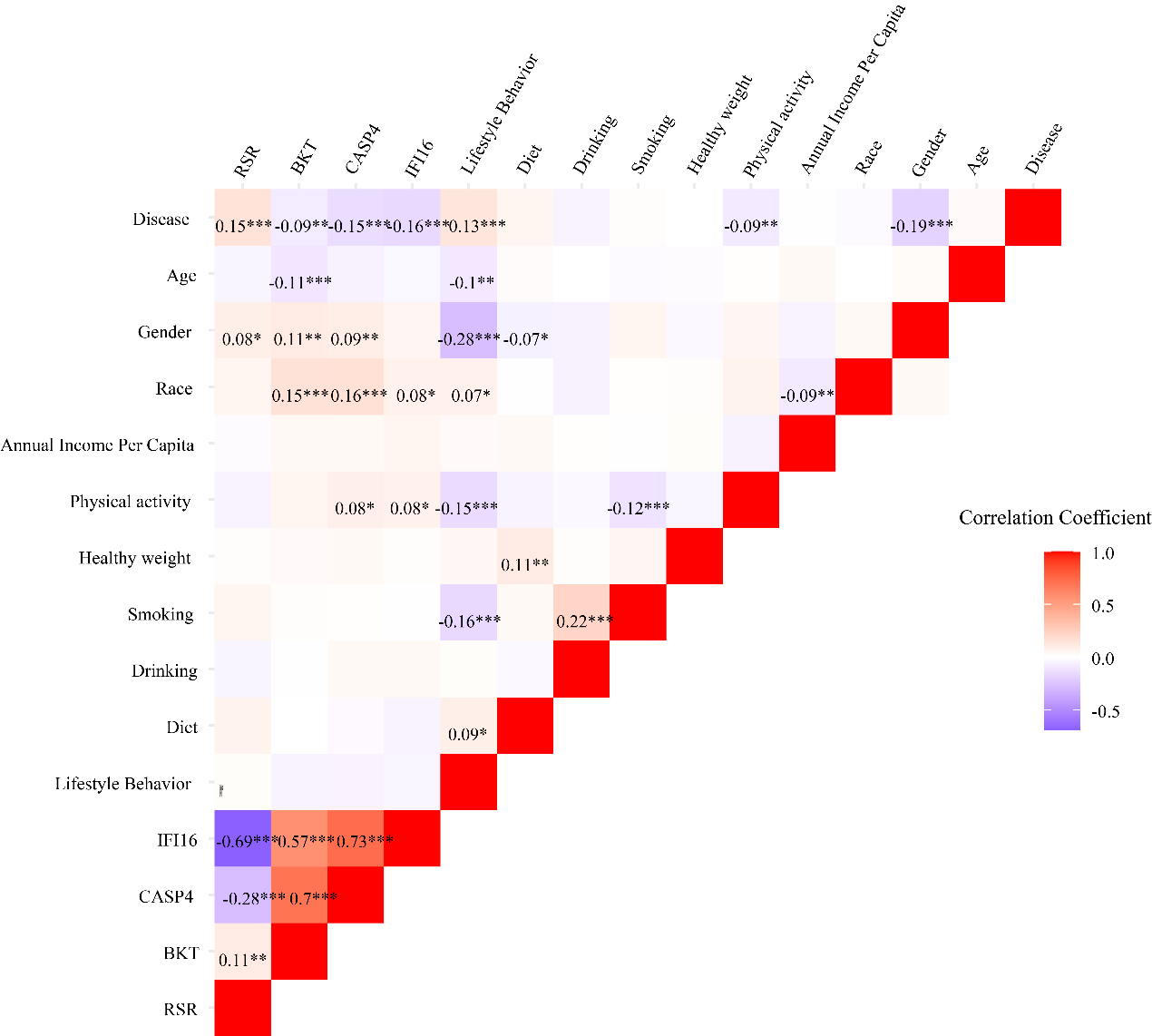
**

*: *P*<0.05;**: *P*<0.01;***: *P*<0.001.

**FigureS2.** Correlation Analysis of Baseline Characteristics, IRDEGs, RSR Score, and Lifestyle with AIT.


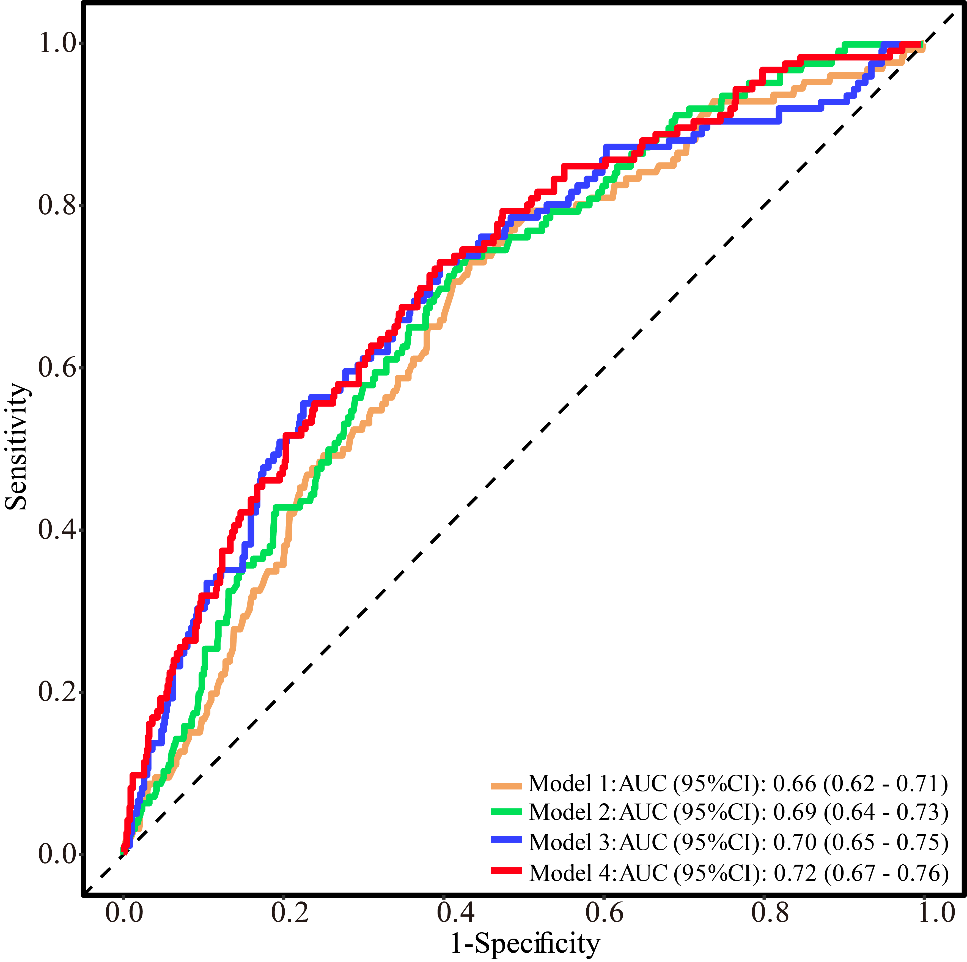


**FigureS3.** Comparison of Predictive Performance Across Variables Using Logistic Regression.

Model 1: age, race, annual income per capita, serum iodine.

Model 2: age, race, annual income per capita, serum iodine, lifestyle behavior.

Model 3: age, race, annual income per capita, serum iodine, RSR.

Model 4: age, race, annual income per capita, serum iodine, lifestyle behavior, RSR.


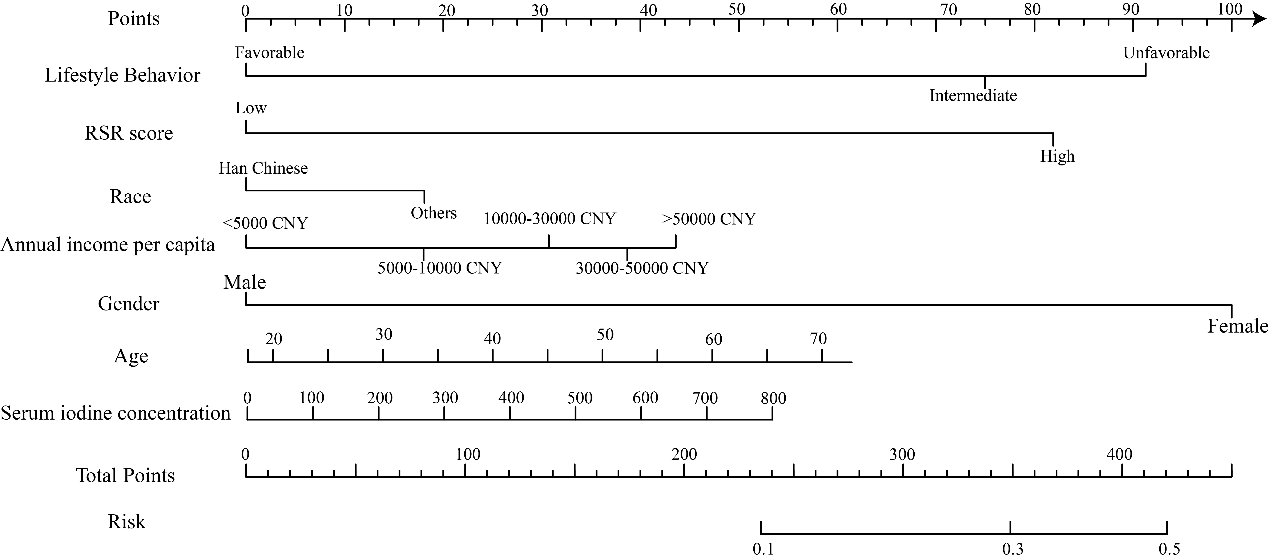


**FigureS4.** Nomogram Derived from the Logistic Regression Model.
